# Fibrin‐specific poly(N‐isopropylacrylamide) nanogels for targeted delivery of tissue‐type plasminogen activator to treat thrombotic complications are well tolerated in vivo

**DOI:** 10.1002/btm2.10277

**Published:** 2021-12-11

**Authors:** Emily P. Mihalko, Kimberly Nellenbach, Manasi Krishnakumar, Nina Moiseiwitsch, Jennifer Sollinger, Brian C. Cooley, Ashley C. Brown

**Affiliations:** ^1^ Joint Department of Biomedical Engineering of University of North Carolina Chapel Hill and North Carolina State University Raleigh North Carolina USA; ^2^ Comparative Medicine Institute North Carolina State University Raleigh North Carolina USA; ^3^ Department of Pathology and Laboratory Medicine University of North Carolina Chapel Hill North Carolina USA

**Keywords:** disseminated intravascular coagulation, fibrin, nanogels, pNIPAM, targeted delivery, thrombosis, tissue plasminogen activator

## Abstract

Targeted drug delivery for maintaining blood fluidity can reduce the risks associated with systemic anticoagulants that can lead to off‐target bleeding. Recently, there has been much interest in targeted delivery of tissue‐type plasminogen activator (tPA) for treating thrombotic complications. The work presented here characterizes a fibrin‐specific nanogel (FSN) design for targeted delivery of tPA to treat thrombotic complications. Fibrin binding and clot degradation were characterized in vitro, and animal models of thrombosis were used to examine nanogel effects on coagulation parameters. In vitro assays showed tPA‐FSNs attach to fibrin in a dose‐dependent manner independent of tPA loading. In animal models of thrombosis, including an electrolytic injury to monitor clot properties in real time, and a lipopolysaccharide‐induced disseminated intravascular coagulation (DIC) animal model, tPA‐FSNs modulated fibrin/fibrinogen and platelet incorporation into clots and at optimized dosing could recover consumptive coagulopathy in DIC. Distribution of unloaded and tPA‐loaded FSNs showed potential clearance of tPA‐FSNs after 24 h, although unloaded FSNs may be retained at sites of fibrin deposits. Maximum tolerated dose studies showed tPA‐FSNs have minimal toxicity up to 20 times the optimized therapeutic dose. Overall, these studies demonstrate the therapeutic efficacy of targeted fibrinolysis for systemic microthrombi and begin to evaluate key translational parameters for tPA‐FSN therapeutics, including optimal tPA‐FSN dosage in a DIC rodent model and safety of intravenous tPA‐FSN therapeutics.

## 1 INTRODUCTION

Maintaining blood fluidity and hemostatic balance is critically important in the vascular system. Initiation of the coagulation cascade can occur as a result of vascular injury, upon which tissue factor‐bearing cells come in contact with blood.^1^, ^2^, ^3^ Additionally, in cases of severe sepsis or systemic inflammatory conditions, the coagulation cascade can also become activated through tissue factor‐mediated pathways.^4^, ^5^, ^6^ While inhibitory pathways and mediators, including fibrinolysis, should normally regulate the overactivation of the coagulation cascade, in conditions like sepsis, inflammation, or trauma, an imbalance of coagulation and fibrinolysis may occur, leading to thrombotic complications.

The activation of the coagulation cascade in vivo culminates in fibrin polymerization, initiated by the serine protease thrombin. Thrombin activates soluble fibrinogen and promotes its polymerization into an insoluble fibrin mesh.^7^ Platelets, also activated by thrombin, incorporate in the forming fibrin mesh creating an immobile clot within the vasculature. Plasmin plays a central role in clot degradation when it becomes activated from fibrin‐bound plasminogen. Plasminogen activators, such as tissue‐type plasminogen activator (tPA), are released into the bloodstream and initiate this process upon binding to fibrin, activating plasminogen, which is transformed to plasmin and cleaves fibrin.^8^ Targeting fibrin for directed thrombolysis therapies represents an optimal strategy because it mimics the body's natural regulation of coagulation.

Dysregulation of clotting or fibrinolysis can result in serious conditions, therefore thrombolytic therapies must be carefully managed. Recombinant tPA is currently approved by the Food and Drug Administration for use in ischemic stroke patients; however, intravenous delivery of tPA can lead to off‐target bleeding. Therefore, there has been much interest in targeted delivery through various strategies, presented in several review articles.^9^, ^10^, ^11^, ^12^, ^13^ In ischemic stroke patients, where targeted delivery of tPA has recently been investigated, approaches include ultrasound‐triggered targeting of thrombolysis with tPA^14^ and magnetic targeting of tPA‐loaded microrods or nanoparticles.^15^, ^16^ Additionally, in events such as myocardial infarction, fibrin‐targeted tPA delivery in conjunction with antifibrotic agent drug delivery has been investigated, showing enhanced cardiac function, potentially due to targeted fibrinolysis of fibrin deposition in injured heart tissue leading to a synergistic effect with antifibrotic agents.^17^ For venous thrombus therapy, tPA delivery through magnetic targeting and ultrasound stimulation has shown success,^18^ and in initial in vitro experimentation and computational simulations, fibrinogen‐mimicking nanovesicles carrying tPA show particle binding to activated platelets and effective clot lysis.^19^ Targeted delivery of tPA could also be promising for more complex disorders such as disseminated intravascular coagulation (DIC). DIC is a secondary disorder to conditions such as sepsis, trauma, or systemic inflammation and leads to excessive thrombin generation creating microvasculature thrombi and eventual clotting factor consumption and hemorrhage.^20^, ^21^, ^22^, ^23^, ^24^, ^25^ In DIC, treatment can be directly opposing; for example, plasma/platelet transfusions are often administered in cases of bleeding, or anticoagulant/fibrinolytic therapies are often administered in cases of thrombosis complications. Further complicating the management of DIC, systemic administration of therapeutics can have deleterious off‐target effects exacerbating the hemostatic imbalance of the disorder.^26^, ^27^, ^28^ Therefore, a targeted approach to deliver thrombolytic agents, such as tPA, to aberrant thrombi also represents a promising treatment strategy for DIC.

To that end, the development of targeted fibrinolytic therapies can play a major role in improving therapeutic options for a variety of thrombotic complications. Fibrin‐targeting therapies offer an attractive strategy to direct treatment to sites of adverse thrombi in the vasculature. Additionally, delivery of plasminogen activators, such as tPA, may help to regulate the overactivation of the coagulation cascade if administered in cases where bleeding phenotypes are not present. To that end, we developed fibrin‐specific nanogels (FSNs), comprised of core‐shell microgels synthesized using precipitation polymerization reactions to make cores containing 90% poly(*N*‐isopropylacrylamide) (pNIPAM) and 10% *N*,*N*′‐methylenebis(acrylamide) (BIS), and shells containing 93% pNIPAM, 2% BIS, and 5% acrylic acid (AAc). Prior to conjugation with any fibrin‐specific element, these particles are referred to as “core‐shell nanogels,” and once conjugated to fragment E fibrin antibody, these particles are referred to as FSNs. FSN carriers are then loaded with tPA to create tPA‐FSNs.

The overall object in this study is to examine tPA‐FSNs action on the dissolution of thrombi in vitro and in vivo and to assess the particles tolerance in vivo. It is hypothesized that tPA‐FSNs work by targeting fibrinolytic capabilities directly to fibrin formations that could help in the dissolution of adverse thrombi and correction of consumptive coagulopathy in circumstances such as DIC, and that tPA‐FSNs are well tolerated in the body even at higher than therapeutic doses. Therefore, tPA‐FSN action was studied in vitro and in vivo to examine fibrin‐binding capabilities, degradation with clots in platelet‐rich and platelet‐poor plasma, the dissolution of thrombi in a DIC rodent model, and coagulation parameters following treatment. Tolerance of tPA‐FSNs at higher than therapeutic doses was studied in healthy mice by monitoring weight, food consumption, serum chemistry, and hematology parameters.

## 2 RESULTS

### 2.1 Nanogel characterization

**FIGURE 1:**
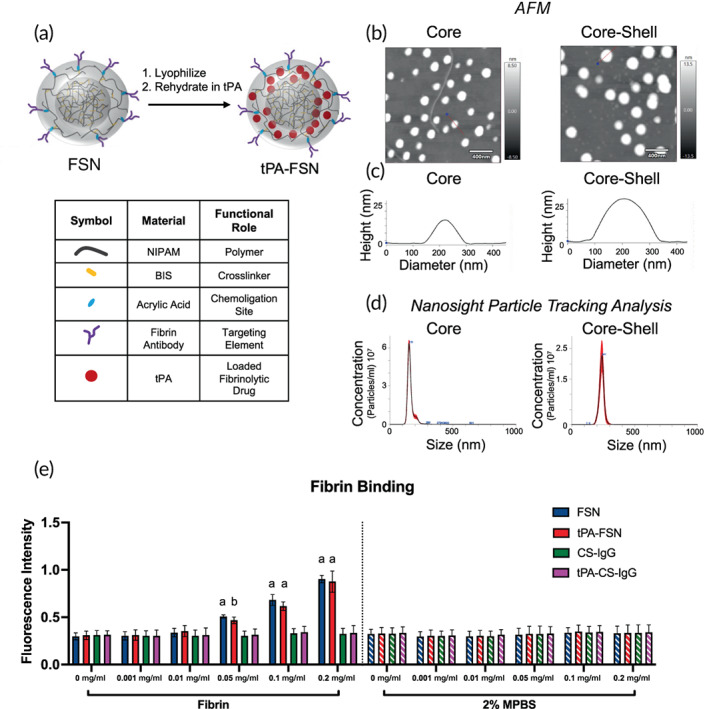
Overview of tissue‐type plasminogen activator‐fibrin‐specific nanogels (tPA‐FSN) particle design and characterization. (a) Core‐shell nanogel composition and schematic of tissue‐type plasminogen activator (tPA) loading into FSNs. (b) Representative atomic force microscopy (AFM) images of core and core‐shell nanogels. (c) Representative height traces of single particles (depicted with a red line through the center) from AFM images of core and core‐shell nanogels. (d) Particle size distribution of hydrodynamic diameter measurements from the core and core‐shell nanogels utilizing NanoSight particle tracking analysis software. At least 10^8^ core and core‐shell nanogels were tracked. (e) Fibrin binding assay of FSNs, tPA‐FSNs, control sheep immunoglobulin G (CS‐IgG) particles, and tPA‐CS‐IgG particles at 0, 0.001, 0.01, 0.05, 0.1, and 0.2 mg/ml concentrations on fibrin‐coated wells or negative control 2% powdered milk in phosphate‐buffered saline (MPBS) wells (*n* = 6–9/group). Mean ± SD is shown. Data were analyzed via a two‐way analysis of variance with a Tukey's post hoc test using a 95% confidence interval. a: *p* < 0.0001 b: *p* < 0.001 compared to IgG control particle types

Fabrication of FSNs (Figure 1a) first was characterized by the successful synthesis of core‐shell (CS) nanogels. Following synthesis via precipitation polymerization reaction, core nanogels were found to have a hydrodynamic diameter of 161 ± 26 nm. Following shell addition, CS nanogels measure 238 ± 19 nm in diameter using NanoSight particle tracking analysis (Figure 1d). After conjugation of CS nanogels to fragment E antibody to create FSNs, particle size was similarly characterized, with a hydrodynamic diameter measuring 230 ± 53 nm, and after loading with tPA, measuring 244 ± 67 nm (Figure S1). A polydispersity index (PDI) of core, CS, FSNs, and tPA‐FSNs was determined as follows: 0.026, 0.0064, 0.053, and 0.075, respectively. Representative atomic force microscopy (AFM) images and height traces are shown and demonstrate a similar increase in particle size following shell addition (Figure 1b,c). Average AFM diameter and height measures from previously studied nanogels of the same formulation are 166 ± 16 nm and 17 ± 3 nm, respectively, for core nanogels, and 263 ± 28 nm and 29 ± 4 nm, respectively, for CS nanogels.^29^ A conjugation efficiency of 74 ± 19% was determined for FSNs (CS particles conjugated to fragment E fibrin antibody). Zeta potential measurements were −4.90 mV for core nanogels, −6.57 mV for CS samples, and −4.07 mV for FSNs, further demonstrating surface coverage. Fibrin binding of FSN and CS nanogels conjugated to non‐binding control sheep immunoglobulin G (CS‐IgG) was analyzed using plate‐based fibrin assays. Compared to CS‐IgG particles, FSNs bind to fibrin in a dose‐dependent manner. Furthermore, minimal binding of FSNs or CS‐IgG particles is seen on negative control wells. Upon tPA loading into both FSNs and CS‐IgG particles, a similar dose‐dependent binding to fibrin is seen with tPA‐FSNs; tPA‐FSNs also showed minimal binding on negative control wells. Minimal differences between fibrin binding with FSNs and tPA‐FSNs were observed on fibrin plates at any concentration. Additionally, no increased binding upon tPA loading in CS‐IgG particles was observed, demonstrating loaded tPA does not play a significant role in particle fibrin binding. A continued increase in fibrin binding potential, up to 0.8 mg/ml, was observed (Figure S3).

### 2.2 tPA‐FSNs degrade clots ex vivo made from human platelet‐poor plasma and platelet‐rich plasma

**FIGURE 2:**
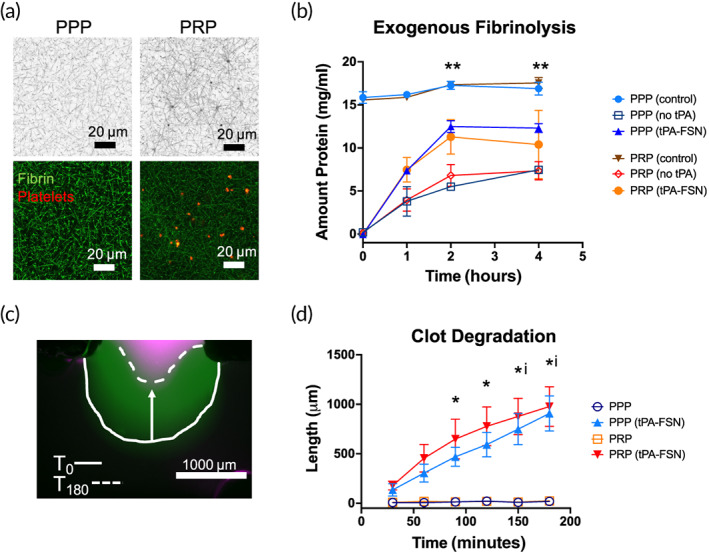
Clot degradation with tissue‐type plasminogen activator‐fibrin‐specific nanogels (tPA‐FSNs) in platelet‐rich and platelet‐poor plasma. (a) Confocal microscopy of platelet‐rich plasma (PRP) and platelet‐poor plasma (PPP) illustrating enhanced fibrin clot structure in platelet‐rich environments compared to platelet‐poor environments. Fibrin fibers are depicted in black (top) and green (bottom), and platelets are shown in red (bottom). (b) Exogenous fibrinolysis assays show enhanced degradation with overlayed tPA‐FSNs compared to no tPA. **p < 0.01 for PPP (no tPA) compared to PPP (tPA‐FSNs). (c) Clot degradation studied in T‐junction fluidic devices is shown as overlayed images at T_0_ (after binding of tPA‐FSNs for 20 min at a wall shear rate of 1 s^−1^) and T_180_ at the stationary clot boundary. (d) Quantification of clot degradation of PPP and PRP stationary clots with and without tPA‐FSNs in flow solutions for 20 min prior to degradation study (*n* = 3 clots per condition). Mean ± SD is shown. * = *p* < 0.05 between PPP conditions. i = *p* < 0.05 between PRP conditions. Data were analyzed via a two‐way analysis of variance with a Tukey's post hoc test using a 95% confidence interval

Clot structure of plasma clots shows enhanced fibrin network with platelet‐rich plasma (PRP) compared to platelet‐poor plasma (PPP) with platelets visualized within the plasma clots of PRP (Figure 2a). PRP platelet count was 362,000 platelets/μl. The action of tPA‐FSN clot dissolution in both PPP and PRP clots was examined using various techniques. Exogenous fibrinolysis was studied by polymerization of both PPP and PRP clots. Clots were overlayed with either tPA‐FSNs or with water containing no tPA as a negative control. Nonpolymerized controls were also included that contained wells of PPP or PRP and no thrombin. At specific time points, protein concentration in the overlay solution depicts the amount of clot degradation. Nonpolymerizing control wells show high levels of protein at all time points. While some increase in protein content was seen in control wells containing no tPA, there was a larger increase in protein content in PPP and PRP wells containing tPA‐FSNs, illustrating the ability of these particles to lyse clots. Statistically significant increases in the amount of protein at 2 and 4 h in the overlayed solutions on PPP containing tPA‐FSNs compared to no tPA were observed. However, no significant increase in protein was observed on PRP clots overlayed with tPA‐FSNs compared to no tPA (*p* = 0.18 at 2 h). This likely reflects the increased stability of platelet‐containing clots compared to platelet‐poor clots.

Since static clot degradation in the exogenous fibrinolysis assays may not recapitulate degradation in vivo, another technique of examining tPA‐FSN clot degradation was through a fluidic T‐junction device. A stationary clot was polymerized either from PPP or PRP. Then, tPA‐FSN solutions were flowed through the perpendicular channel over the stationary clot boundary site for 20 min prior to monitoring degradation of the clot boundary over the course of 3 h. While control solutions of PPP or PRP did not show any clot degradation over the course of 3 h, tPA‐FSN containing flow solutions did show significant degradation within that time frame. Additionally, no significant difference between PPP and PRP clot degradation was observed, demonstrating the ability of tPA‐FSNs to act on various clot densities to enable clot dissolution.

### 2.3 tPA‐FSNs modulate fibrin and platelet incorporation in an electrolytic injury thrombosis model

**FIGURE 3:**
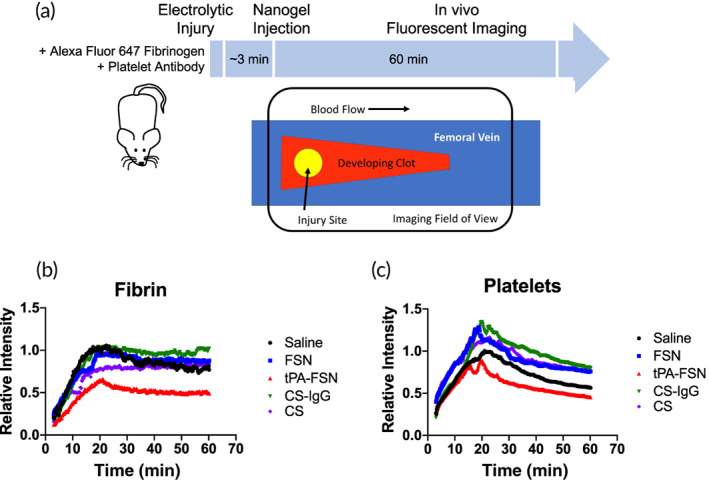
Clot dynamics in vivo with tissue‐type plasminogen activator (tPA)‐loaded nanogels. (a) Schematic of electrolytic injury induced in mice followed by nanogel injections (~3 min after injury) and intravital fluorescent imaging continuously monitoring fibrin/fibrinogen and platelet recruitment at the injury site up to 60 min post‐injury. Treatment groups include saline, fibrin‐specific nanogels (FSNs), tPA‐FSNs, CS nanogels, and control sheep immunoglobulin G (CS‐IgG) nanogels (*n* = 4 animals/group). (b) Intravital microscopy quantification demonstrates accumulation of fibrin/fibrinogen at the injury site with less fibrin incorporation upon tPA‐FSN treatment. (c) Intravital microscopy quantification also demonstrates accumulation of platelets at the injury site and similarly shows reduced platelet incorporation with tPA‐FSNs. Data presented are mean fluorescent intensity plots over time (frame rate = 0.167 min)

To assess the accumulation and dissolution of fibrin at sites of thrombus formation in the presence of tPA‐FSNs, intravital microscopy was used with a well‐established rodent electrolytic injury model. Following injury, treatments were administered (3 min post‐injury), and fibrin/fibrinogen and platelet recruitment were monitored at the injury site continuously for 60 min post‐injury using intravital microscopy (frame rate = 0.167 min). The accumulation of fibrin/fibrinogen is observed in the electrolytic injury mouse model for all nanogel groups, including saline, FSNs, tPA‐FSNs, CS, and CS‐IgG nanogels. Although, tPA‐FSN treatment resulted in a lower peak relative intensity of fibrin/fibrinogen at the thrombus site, and a reduced initial slope, compared to control conditions (saline, FSNs, CS‐IgG, and CS particles; Figure 3b). Similarly, while platelets accumulate at the site of thrombus for all groups, animals receiving tPA‐FSNs reached a lower relative intensity of platelets incorporated compared to controls (Figure 3c). FSNs and CS particles may enhance platelet recruitment due to their influence on platelet margination; however, future studies are needed to determine this mechanism. These data suggest that following the initiation of thrombus formation, tPA‐FSNs can modulate the accumulation of fibrin and platelets, illustrating their potential efficacy in disease states of hypercoagulation.

### 2.4 tPA‐FSNs mitigate thrombi complications in a DIC rodent model at an optimal dose

**FIGURE 4:**
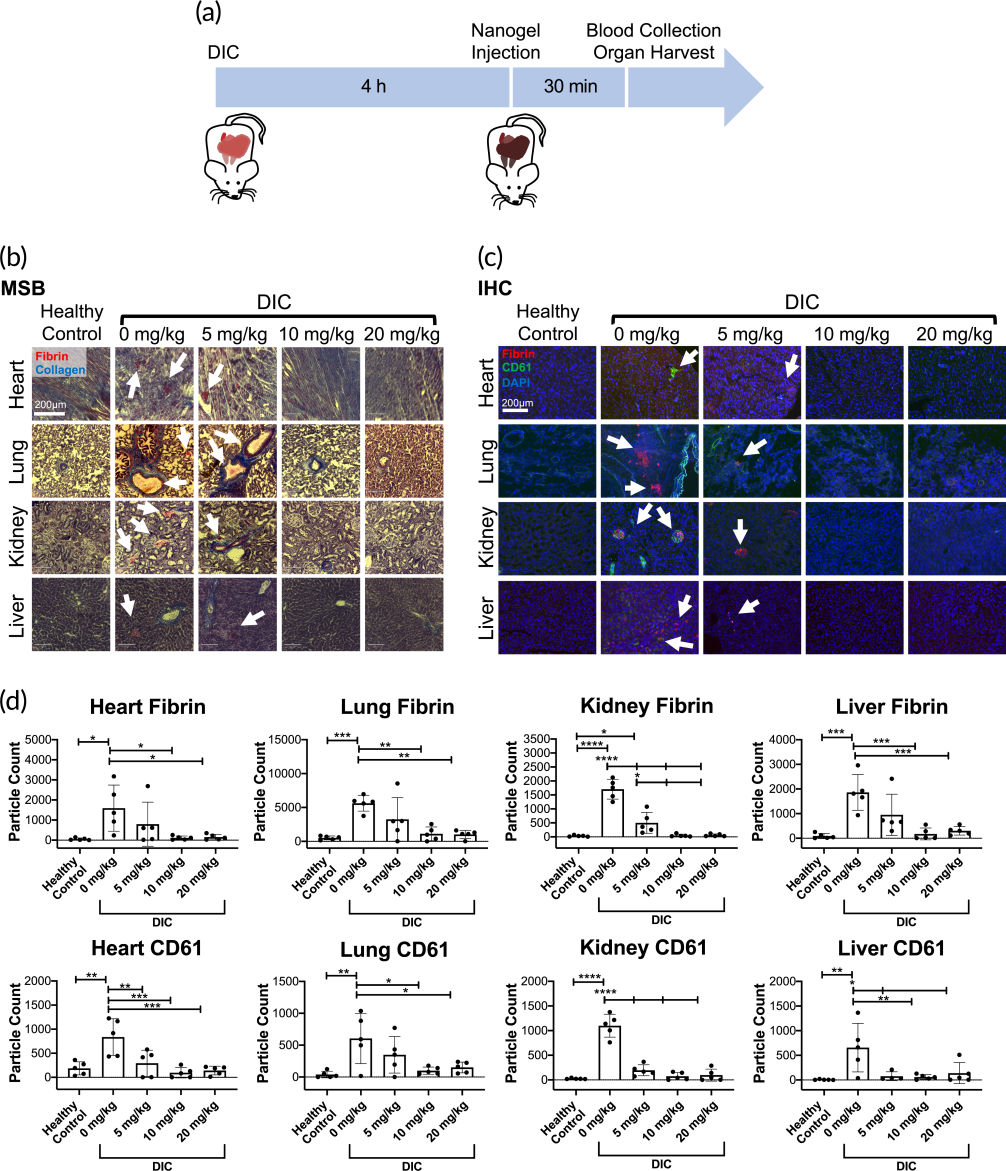
Dose‐response of tissue‐type plasminogen activator‐fibrin‐specific nanogel (tPA‐FSN) treatment and thrombi presentation in a disseminated intravascular coagulation (DIC) rodent model. (a) Schematic of the DIC animal model used including treatment and terminal endpoints. (b) Martius scarlet blue (MSB) stained tissue sections of heart, lung, kidney, and liver from control animals and DIC animals treated with 0, 5, 10, and 20 mg/kg tPA‐FSNs (fibrin = red, collagen = blue). (c) Immunohistochemistry (IHC) tissue sections of heart, lung, kidney, and liver from control animals and DIC animals treated with 0, 5, 10, and 20 mg/kg tPA‐FSNs (fibrin = red, CD61 = green, DAPI = blue). (d) Quantification of IHC fibrin and platelet (CD61) presentation in tissue sections via ImageJ particle count analysis. For each organ, at least three quantified tissue sections were measured and averaged per animal. The mean of five animals per group ± SD is shown. Data were analyzed via a one‐way analysis of variance with a Tukey's post hoc test using a 95% confidence interval. *p < 0.05, **p < 0.01, ***p < 0.001, ****p < 0.0001

To assess the ability of tPA‐FSNs to mitigate thrombotic complications, a lipopolysaccharide (LPS)‐induced DIC rodent model was used and a dose‐response of tPA‐FSNs was examined and compared to control animals not receiving LPS or treatment. Heart, lung, kidney, and liver tissue sections, which were stained with Martius scarlet blue (MSB), show fibrin deposition in all DIC organs that were untreated (Figure 4a). While substantial fibrin deposition was also visualized at 5 mg/kg tPA‐FSN dose in the DIC animals, at 10 and 20 mg/kg, the appearance of fibrin deposition appears to be reduced in the heart, lung, kidney, and liver. To quantify the decrease in thrombi, immunohistochemistry (IHC) images that were stained for fibrin and platelets (CD61) were imaged and quantified using ImageJ software for fibrin or platelet particle counts (Figure 4b,c). In control animals, minimal amounts of fibrin or platelets (CD61) were observed in the heart, lung, kidney, and liver. In DIC animals receiving 0 mg/kg tPA‐FSNs, significant increases in fibrin and platelet presentation were observed in all organs. At 5 mg/kg tPA‐FSN doses in DIC animals, some significant reduction in fibrin deposition was observed in the kidney compared to 0 mg/kg, although not in the other organ types examined. At 5 mg/kg, significant reductions in platelets (CD61) sequestered in the organs were observed in the heart, kidney, and liver compared to 0 mg/kg. At both 10 and 20 mg/kg tPA‐FSN in DIC animals, significant reductions in both fibrin and platelets (CD61) in the organs were observed compared to 0 mg/kg, demonstrating the capabilities of these nanogels to reduce the number of sequestered platelets and microthrombi presentations that are observed in this animal model of DIC.

**FIGURE 5:**
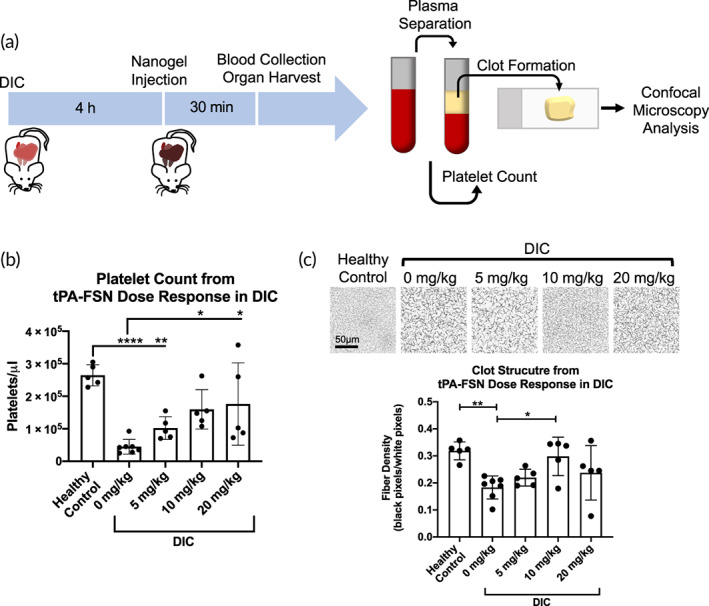
Platelet count dose‐response and clot structure measured after tissue‐type plasminogen activator‐fibrin‐specific nanogel (tPA‐FSN) treatment in a disseminated intravascular coagulation (DIC) rodent model. (a) Schematic of the DIC animal model used in addition to the treatment timing and terminal endpoints. Terminal blood collection was followed by platelet counts and plasma separation to form ex vivo clots and examine clot structure at the study endpoint. (b) Manual platelet counts were conducted for control and DIC animals treated with 0, 5, 10, and 20 mg/kg tPA‐FSNs. (c) Confocal microscopy images were taken, and fiber density quantification was conducted of clots polymerized from isolated platelet‐poor plasma from control animals and DIC animals treated with 0, 5, 10, and 20 mg/kg tPA‐FSNs. Clots from each animal were polymerized and three images per clot were quantified for fiber density and averaged. *n* = at least 5 animals/group. Mean ± SD is shown. Data were analyzed via a one‐way analysis of variance with a Tukey's post hoc test using a 95% confidence interval. *p < 0.05, **p < 0.01, ****p < 0.0001

Platelet counts from blood samples drawn 30 min following tPA‐FSN treatment show significant reductions in platelet counts in the DIC animals treated with 0 mg/kg tPA‐FSNs compared to control animals, matching clinical presentations of the disorder (Figure 5a). At 5 mg/kg tPA‐FSNs in the DIC animals, a significant reduction in platelet count was still observed. However, at 10 and 20 mg/kg tPA‐FSN treatment in DIC animals, a significant increase compared to 0 mg/kg doses was observed. These results indicate potential recovery of the consumptive coagulopathy phenotype of the disorder. Isolated PPP from the blood samples taken from each animal was also examined for clot structure via confocal microscopy (Figure 5b). Compared to control animals, DIC animals with 0 mg/kg tPA‐FSNs show consumptive coagulopathy with a significantly reduced fibrin clot network. At 10 mg/kg tPA‐FNS treatment, clot structure was significantly improved. However, the improvement in clot structure was not sustained at higher doses of 20 mg/kg tPA‐FSNs, perhaps due to increased tPA and hyperfibrinolysis in the samples. These results demonstrate an optimal dosage at 10 mg/kg will reduce thrombi presentation, release sequestered clotting factors, and improve consumptive coagulopathy in our DIC model.

### 2.5 Distribution of nanogels in a DIC rodent model correlate to fibrin thrombi in tissue and to tPA‐FSN mode of action

**FIGURE 6:**
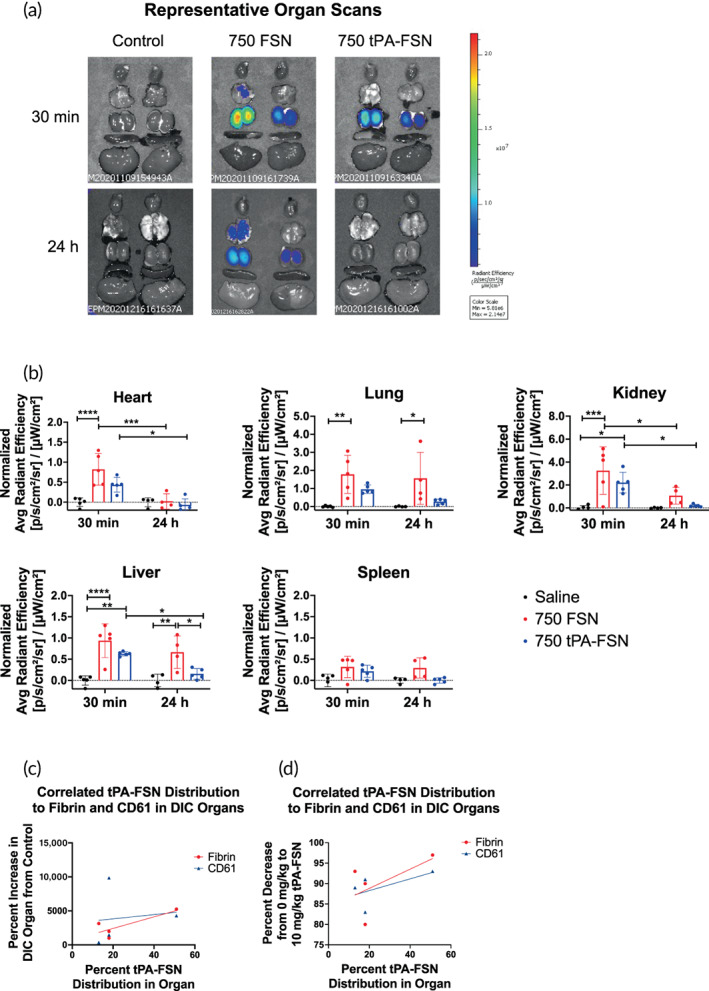
Distribution of unloaded fibrin‐specific nanogels (FSNs) and tissue‐type plasminogen activator‐FSNs (tPA‐FSNs) in a disseminated intravascular coagulation (DIC) rodent model 30 min and 24 h after injection. (a) Representative IVIS organ scans from DIC animals receiving saline (control), 10 mg/kg FSNs, or 10 mg/kg tPA‐FSNs labeled with 750 Vivo Tag for either 30 min or 24 h prior to organ harvest. (b) Average radiant efficiency was measured from each organ and quantification of particle distribution was normalized to control organs. *n* = 4–5 animals per group. Mean ± SD is shown. Data were analyzed via a two‐way analysis of variance with a Tukey's post hoc test using a 95% confidence interval. *p < 0.05, **p < 0.01, ***p < 0.001, ****p < 0.0001. (c) Correlated tPA‐FSN distribution at 30 min to the percent increase of either fibrin or platelets (CD61) in DIC organs compared to control organs show a positive relationship that tPA‐FSNs accumulate to fibrin deposition sites. (d) Correlated tPA‐FSN distribution at 30 min to the percent decrease of either fibrin or platelets (CD61) in DIC organs treated with 10 mg/kg tPA‐FSNs compared to 0 mg/kg tPA‐FSNs show that distribution and tPA‐FSNs efficacy to lyse clots are positively correlated

In the DIC rodent model, after 30 min, FSNs and tPA‐FSNs both show significant amounts of nanogel accumulation in the kidney and liver compared to control animals that received saline injections (Figure 6a,b). FSNs also accumulate in the lung and the heart, however, a significant increase in tPA‐FSN accumulation was not observed compared to control animals receiving saline. After 24 h, a significant amount of FSN particles was still present in the lung and liver compared to saline controls, but a significant reduction in the amount of FSNs at 24 h compared to 30 min in the kidney demonstrates the unloaded FSN particles may be cleared over longer time scales. However, since distribution studies were conducted in DIC‐induced animals, it is likely thrombi persisted in the organs after 24 h and sequestered FSNs at sites of fibrin deposition, thereby limiting clearance. With tPA‐FSNs, however, after 24 h, a significant reduction in the accumulation of particles was apparent in the kidney, heart, and liver compared to particle distribution at 30 min. Furthermore, no significant amounts of tPA‐FSN particles were observed in the heart, lung, kidney, liver, or spleen after 24 h compared to control animals receiving saline. These data show that tPA‐FSNs can be cleared from the body, potentially because they allow for thrombi dissolution in the DIC model and therefore do not become sequestered in fibrin depositions in the organs.

The percent of tPA‐FSN distribution in the heart, lung, kidney, and liver was measured by the ratio of the average radiant efficiency per organ over the sum of the average radiant efficiency in those organs. Thus, it was found that 13% was in the heart, 18% was in the lung, 51% was in the kidney, and 18% was in the liver. The percent increase of fibrin or platelets (CD61) in DIC organs receiving 0 mg/kg treatment compared to control organs was plotted against the percent of tPA‐FSNs in the organs at 30 min (Figure 6c). A simple linear regression gives a best‐fit line with a positive correlation of fibrin increases in the organ to tPA‐FSN distribution shows that particles may be accumulating at early time points in organs where the most fibrin deposition is present. The percent decrease of fibrin or platelets (CD61) in DIC organs receiving 10 mg/kg tPA‐FSNs compared to 0 mg/kg was also plotted against the percent of tPA‐FSNs in the organs at 30 min (Figure 6d). A simple linear regression again gives a best‐fit line with a positive slope showing a positive correlation of fibrin and platelets (CD61) decrease in the organs to tPA‐FSN distribution, indicating the more particles present in the organ, the more clot dissolution can occur.

### 2.6 Analysis of toxicity of tPA‐FSNs in vivo at supratherapeutic doses

**FIGURE 7:**
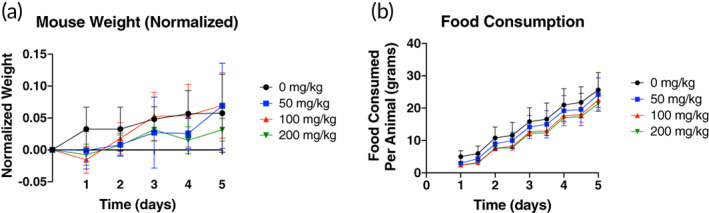
Tolerance of tPA‐FSNs in healthy mice. (a) After receiving 0, 50, 100, or 200 mg/kg tissue‐type plasminogen activator‐FSN (tPA‐FSN), healthy mice were weighed daily for 5 days and animal weight was normalized to their starting weight. (b) Food consumption was monitored via a change in food weight of the cage divided by the number of animals in the cage and measured for 5 days after tPA‐FSN injection. *n* = 5 animals/group. Mean ± SD is shown. Data were analyzed via a two‐way analysis of variance with a Tukey's post hoc test using a 95% confidence interval

At doses, 5–20 times the optimized therapeutic dose of 10 mg/kg, administration in healthy mice shows no significant changes in animal weight between groups up to 5 days after injection. (Figure 7a). Additionally, no significant differences in food consumption between treatment groups were observed over 5 days (Figure 7b). Hematology parameters, including neutrophils, reticulocytes, white blood cells, red blood cells, monocytes, and platelet count (Table 1), measured at the study endpoint at 5 days postinjection show no significant differences between treatment groups and controls. Serum chemistry parameters, also measured at the study endpoint at 5 days postinjection, show significant decreases in blood urea nitrogen at 10 and 20 mg/kg compared to control animals, which could indicate effects on liver function; however, alanine aminotransferase, alkaline phosphatase, aspartate aminotransferase, total bilirubin, albumin, and total protein show no significant differences in the 10 and 20 mg/kg groups compared to control animals. Additionally, potassium levels at 50 mg/kg are significantly higher than controls, although the levels measured fall within the normal range.^30^ The degree of hemolysis caused by nanogel exposure is shown in Figure S2. At the human equivalent dose (HED) of 0.01 mg/ml, and 100× the HED of 1 mg/ml, CS nanogels, FSNs, and tPA‐FSNs are all below 5% hemolysis.

**TABLE 1:** Maximum tolerated dose hematology and serum chemistry parameters

Hematology parameters				
	tPA‐FSN Dose			
	0 mg/kg	50 mg/kg	100 mg/kg	200 mg/kg
Neutrophil (%)	6.3 ± 1.7	8.1 ± 6.7	7.0 ± 2.0	7.0 ± 0.9
Neutrophil (μl)	469 ± 51	307 ± 91	549 ± 179	627 ± 533
Reticulocyte (%)	4.2 ± 0.5	4.0 ± 0.4	4.0 ± 0.8	4.4 ± 1.0
WBC (K/μl)	7.8 ± 1.9	5.9 ± 3.3	7.8 ± 0.8	5.9 ± 2.0
Absolute reticulocyte (K/μl)	403 ± 46	366 ± 62	387 ± 79	421 ± 95
RBC (M/μl)	9.65 ± 0.52	9.24 ± 1.07	9.71 ± 0.31	9.51 ± 0.70
HGB (g/dl)	14.1 ± 0.8	13.6 ± 1.4	14.2 ± 0.3	14.0 ± 0.6
Lymphocyte (/μl)	6979 ± 1804	5340 ± 3220	6747 ± 662	4946 ± 1854
Lymphocytes (%)	89.5 ± 1.7	84.6 ± 11.9	86.6 ± 2.0	83.3 ± 6.3
HCT (%)	45.9 ± 1.9	44.1 ± 3.9	45.2 ± 1.0	45.2 ± 3.0
Monocyte (μl)	192 ± 91	80 ± 15	279 ± 122	178 ± 108
Monocytes (%)	2.4 ± 0.9	1.1 ± 0.2	3.5 ± 1.3	3.3 ± 2.2
Eosinophil (μl)	129 ± 43	196 ± 113	212 ± 90	134 ± 97
Eosinophils (%)	1.6 ± 0.4	3.5 ± 1.9	2.8 ± 1.3	2.8 ± 0.1
MCV (fl)	47 ± 1	48 ± 2	47 ± 1	47 ± 0
Basophil (μl)	12 ± 12	9 ± 1	13 ± 11	14 ± 11
Basophils (%)	0.1 ± 0.1	0.5 ± 0.6	0.2 ± 0.1	0.3 ± 0.4
MCH (pg)	14.6 ± 0.2	14.7 ± 0.3	14.7 ± 0.3	14.8 ± 0.5
MCHC (g/dl)	30.7 ± 0.6	30.8 ± 0.5	31.5 ± 0.8	31.1 ± 1.1
Platelet count (K/μl)	1034 ± 189	916 ± 423	999 ± 379	1080 ± 451

Serum chemistry parameters				
	tPA‐FSN dose			
	0 mg/kg	50 mg/kg	100 mg/kg	200 mg/kg
ALP (U/L)	77 ± 12	66 ± 19	63 ± 11	61 ± 4
AST (U/L)	73 ± 27	95 ± 54	71 ± 20	45 ± 13
ALT (U/L)	26 ± 3	28 ± 2	31 ± 7	23 ± 3
Creatine kinase (U/L)	213 ± 69	439 ± 272	548 ± 271	182 ± 92
Albumin (g/dl)	2.9 ± 0.1	2.8 ± 0.2	2.9 ± 0.1	2.9 ± 0.3
Total bilirubin (mg/dl)	0.2 ± 0.1	0.2 ± 0.0	0.2 ± 0.0	0.2 ± 0.0
Bilirubin—unconjugated (mg/dl)	0.2 ± 0.1	0.2 ± 0.0	0.2 ± 0.0	0.2 ± 0.0
Bilirubin—conjugated (mg/dl)	0.0 ± 0.0	0.0 ± 0.0	0.0 ± 0.0	0.0 ± 0.0
Total protein (g/dl)	4.7 ± 0.2	4.5 ± 0.2	4.6 ± 0.1	4.8 ± 0.6
Globulin (g/dl)	1.8 ± 0.1	1.7 ± 0.1	1.7 ± 0.1	1.9 ± 0.2
BUN (mg/dl)	32 ± 4	30 ± 2	27 ± 1*	27 ± 2*
Creatinine (mg/dl)	0.1 ± 0.0	0.1 ± 0.0	0.1 ± 0.1	0.1 ± 0.1
Cholesterol (mg/dl)	75 ± 10	79 ± 4	78 ± 8	92 ± 26
Glucose (mg/dl)	250 ± 41	245 ± 30	246 ± 9	263 ± 20
Calcium (mg/dl)	9.6 ± 0.1	9.3 ± 0.2	9.4 ± 0.3	9.4 ± 0.5
Phosphorus (mg/dl)	6.7 ± 0.5	6.7 ± 0.6	6.5 ± 0.7	8.4 ± 2.2
Bicarbonate TCO2 (mmol/L)	16 ± 0	15 ± 2	15 ± 1	14 ± 4
Chloride (mmol/L)	110 ± 3	110 ± 2	108 ± 1	108 ± 1
Potassium (mmol/L)	4.2 ± 0.2	5.0 ± 0.6*	4.4 ± 0.4	4.5 ± 0.2
ALB/GLOB ratio	1.6 ± 0.1	1.6 ± 0.1	1.7 ± 0.1	1.6 ± 0.1
Sodium (mmol/L)	151 ± 3	149 ± 2	150 ± 1	148 ± 3
BUN/creatinine ratio	316.0 ± 41.0	265.0 ± 62.8	161.0 ± 110.5*	217.0 ± 75.0
NA/K ratio	36 ± 2	30 ± 4*	35 ± 4	33 ± 1

*Note*: After receiving 0, 50, 100, or 200 mg/kg tPA‐FSNs, blood samples from each animal were analyzed for serum chemistry and hematology parameters, listed above. *n* = 4–5 animals/group depending on available sample volume. Mean ± SD is shown. Data were analyzed via a one‐way analysis of variance with a Dunnett post hoc test using a 95% confidence interval.

Abbreviations: ALT, alanine transaminase; ALP, alkaline phosphatase; AST, aspartate transaminase; BUN, blood urea nitrogen; HCT, hematocrit; HGB, hemoglobin; MCH, mean corpuscular hemoglobin; MCHC, mean corpuscular hemoglobin concentration; MCV, mean corpuscular volume; RBC, red blood cells; tPA‐FSN, tissue‐type plasminogen activator‐fibrin‐specific nanogel; WBC, white blood cells.

* *p* < 0.05.

## 3 DISCUSSION

Thrombotic disorders are a serious complication associated with a variety of diseases including ischemia‐reperfusion injury, organ transplant rejection, trauma, and sepsis.^31^ With the intersection of the hemostatic and inflammatory pathways, thrombotic complications can arise in a broad range of human disorders. The drastic imbalance of blood fluidity in these disease states can lead to significant increases in mortality, especially in cases such as DIC where mortality in sepsis patients with DIC is twice as high as patients without DIC, and up to 50% of cases of sepsis can lead to DIC.^26^, ^32^

Consequently, in treating these thrombotic disorders and coagulopathies, studies have examined a variety of thrombolytic and anticoagulant therapies, although bleeding remains an underlying issue, as coagulation and fibrinolysis remain a challenge to balance.^31^ Targeted delivery of fibrinolytic therapies to treat thrombosis can reduce the amount of circulating fibrinolytic agent in the bloodstream to reduce the risks of bleeding in these patient populations. However, in events such as DIC with systemic coagulopathy, targeted therapies with ultrasound or magnetic targeting of drug delivery are not a feasible option. Thus, utilizing the coagulation cascade components as the targeting element for the delivery of fibrinolytic agent is a promising strategy that does not require external guidance. This delivery strategy could also reduce the effective therapeutic dose of tPA, reducing the inherent bleeding risk of fibrinolytic administration. Indeed, even assuming 100% loading efficiency, the amount of tPA delivered via FSNs would be 1.45 μg/mg of FSNs with the above loading conditions, and at the 20 mg/kg tPA‐FSN dose examined here, 0.029 mg/kg tPA would be delivered, which is ~30 times less than the therapeutic dose for stroke (0.9 mg/kg) yet more than the endogenous tPA concentrations of 0.1 nM.^27^ Future studies should examine the exact delivered concentrations of tPA in vivo within the plasma and injury sites. Additionally, especially in DIC where consumptive coagulopathy is a critical issue, targeted dissolution of aberrant microthrombi can help to release sequestered clotting factors. Here, we show that at optimal doses of tPA‐FSNs, it is possible to reduce the number of thrombi and release sequestered clotting factors to a point where a consumptive coagulopathy may be recovered, illustrated here through improved plasma clot structure ex vivo. Previously published results studying tPA‐FSN action in this DIC model showed that improvements in ex vivo plasma clot structure translated to reductions in bleeding in a liver laceration injury.^29^

The timing of these therapeutics to treat thrombosis complications associated with disorders is a critical consideration. While these studies have shown the efficacy of tPA‐FSNs as a therapeutic to lyse aberrant thrombi, prophylaxis treatment was not examined. Future studies should examine the efficacy of these therapeutics on a time scale of administration and compare it to free tPA. While nontargeted tPA delivery did not improve DIC outcomes when studied previously compared to tPA‐FSNs, prophylaxis administration may differ and should be a consideration in the design of future studies. These studies provide valuable insight into the treatment of thrombotic complications associated with sepsis, especially since sepsis‐induced coagulopathy and DIC diagnostic criteria can direct treatment clinically as the disorder progresses.

Consideration of particle clearance is important, because the circulation of these therapeutics could have negative effects on blood fluidity, especially following the delivery of tPA, if particles are not able to be cleared from the body. In our distribution studies of these particles in a DIC model, we see tPA‐FSN clearance after 24 h; however, unloaded FSNs may be sequestered in the organs by binding to fibrin. Future studies are required to fully examine the mechanism of clearance of these particles. The safety of these therapeutics though, examined in healthy animals, appears to show limited toxicity upon the administration of 20× the optimized therapeutic dose of tPA‐FSNs, and no significant change in hematology parameters in these animals is a promising result indicating that the particles alone do not drastically influence blood fluidity. Future studies should also examine inflammatory levels in the blood postinjections to determine potential unintended peripheral immune activation.

## 4 MATERIALS AND METHODS

### 4.1 Nanogel synthesis and characterization

CS pNIPAM (Sigma–Aldrich) nanogels were synthesized using precipitation polymerization reactions as previously published.^29^ Briefly, core nanogels were synthesized in a 140 mM reaction with 90% pNIPAM and 10% *N*,*N*′‐methylenebis(acrylamide) (BIS) (Sigma‐Aldrich) with 2 mM sodium dodecyl sulfate (SDS), initiated with 2 mM ammonium persulfate (APS). Following purification of core nanogels, a 40 mM shell synthesis reaction contained 93% NIPAM, 2% BIS, 5% AAc, 0.5 mM SDS, and 40% by volume core nanogel solution was initiated with 1.0 mM APS. Nanogels were purified by dialysis against ultrapure water over 72 h. FSNs were synthesized by conjugating a sheep antihuman fibrin fragment E polyclonal antibody (Affinity Biologicals) to CS nanogels using 1‐ethyl‐3‐(3‐dimethylaminopropyl) carbodiimide/sulfo *N*‐hydroxysuccinimide (EDC/sulfo‐NHS) chemistry. EDC/sulfo‐NHS conjugation allows for coupling of particles to available amine‐reactive groups on selected binding motifs, and the fibrin fragment E antibody was chosen specifically because it is readily available and allows for detection via secondary methods. A non‐binding sheep IgG isotype (Thermo Fisher Scientific) was conjugated to CS nanogels as a control for binding studies. Nanogel size was characterized via NanoSight particle tracking analysis (Malvern Panalytical) and AFM (Asylum Research). PDI was calculated from NanoSight particle tracking analysis using the following equation: PDI = (σ/*d*)^2^, where σ is the SD of particle distribution and *d* is the average hydrodynamic particle diameter. Zeta potential was tested for core, CS, and FSN particles. Conjugation efficiency of the fragment E fibrin antibody to CS nanogels was determined utilizing a known particle/ml concentration (NanoSight) and a CBQCA protein quantitation kit (Thermo Fisher Scientific C6667) to determine the amount of antibody per mg FSN, which taken together give the amount of antibody per FSN particle. Based on the known amount of antibody and CS particles added to the conjugation reaction, a conjugation efficiency was calculated.

tPA (Sigma Aldrich) was loaded into FSNs using a rehydration “breathing in” technique where lyophilized nanogels were rehydrated in a tPA solution (29 μg/ml) at 20 mg/ml.^33^, ^34^ After agitation at 4°C for 24 h to allow for drug entanglement into the polymer network of the nanogels, tPA‐FSNs were centrifuged to remove unloaded tPA, washed with ultrapure water, and lyophilized for future use.

To examine fibrin binding capabilities of the particles, fibrin attachment assays were conducted using a plate‐based assay with samples of FSNs, tPA‐FSNs, CS nanogels conjugated to isotype control antibodies (CS‐IgG), and tPA‐CS‐IgG. Thin fibrin layers were deposited using a previously published method^35^ into wells of a 96 well plate and blocked with 2% MPBS using an adapted protocol.^36^, ^37^ Negative control wells were incubated with 2% MPBS for at least 3 h at room temperature and rinsed with HEPES buffer (0.025 M HEPES, 0.15 M NaCl, 5 mM CaCl_2_, pH 7.4). Nanogel samples were incubated on prepared fibrin or MPBS wells for 1 h followed by wash steps, incubation with 10 μg/ml fluorescein isothiocyanate conjugated anti‐sheep secondary antibody (Fisher Scientific 31627), and final wash steps. Fluorescent readings (ex: 485 em: 538) were obtained (Thermo Fisher Scientific: Fluoroskan Ascent). Assays were conducted in triplicate.

### 4.2 Clot degradation with tPA‐FSNs using PRP and PPP

To examine tPA‐FSN clot degradation in both platelet‐rich and platelet‐poor conditions, human PRP was obtained from New York Blood Center. Platelet concentration was manually counted using a hemocytometer. Separation of PPP was conducted through centrifuging PRP at 4000×*g* for 6 min. To first analyze differences in clot network properties, clots formed from PRP and PPP were examined using confocal microscopy. Clots were made with 0.5 U/ml human alpha thrombin (Thermo Fisher Scientific), 0.05 mg/ml Alexa‐Fluor 488 fibrinogen (Thermo Fisher Scientific), in buffer containing 0.025 M HEPES, 0.15 M NaCl, 5 mM CaCl_2_. Imaging was conducted on a Zeiss Laser Scanning Microscopy (LSM 710, Zeiss Inc., White Plains, NY) after at least 2 h to allow for polymerization. Then 1.89 μm z‐stack images were taken. Platelets were stained using Vybrant DiI Cell‐Labeling Solution (Thermo Fisher Scientific). Binary z‐stack projections are shown in addition to fluorescent images.

Exogenous fibrinolysis assays were conducted to examine clot degradation of PPP and PRP clots in the presence of tPA‐FSNs. In a 96 well plate, 80 μl clots were polymerized with 0.5 U/ml thrombin, 0.025 M HEPES, 0.15 M NaCl, and 5 mM CaCl_2_ for 6 h. Control wells with unpolymerized PPP or PRP were used. 80 μl solutions were overlayed on the clot with or without tPA‐FSNs (1 mg/ml). At specific time points, aliquots were taken off the overlayed solution and measured for fibrinogen content using a Thermo Scientific NanoDrop Spectrophotometer (extinction coefficient 15.1). For each condition, three duplicate clots were measured for degradation.

To examine clot degradation under flow, a custom‐made polydimethylsiloxane (PDMS) fluidic device was utilized where a T‐junction clot reservoir allows a stationary clot boundary and perpendicular flow of nanogel‐containing solution. Clots containing PPP or PRP with 0.5 U/ml thrombin, 0.05 mg/ml Alexa‐Fluor 488 fibrinogen, and 0.025 M HEPES, 0.15 M NaCl, and 5 mM CaCl_2_ were pipetted into the clot reservoir prior to polymerization. Clots were allowed to fully polymerize for at least 2 h prior to experimentation. Following polymerization, the fluidic device was placed on an EVOS FL Auto Imaging System, and solutions containing PPP or PRP with or without tPA‐FSNs (1 mg/ml) were flowed into the device at physiologically similar wall sheer rate (WSR) of 1 s^−1^ for 20 min. Chosen WSR was determined based on physiologically similar values and available sample volumes.^38^ Time‐lapse images were obtained and after 20 min, the flow was stopped. Degradation of the clots was examined for 3 h with time‐lapsed images obtained every 10 min. T0 indicates the start of monitoring degradation immediately following 20 min of flow. Images were analyzed using ImageJ to measure clot degradation over time by quantifying the distance of the clot boundary at distinct time points compared to T0. Three fluidic experiments were conducted per condition.

### 4.3 Fibrin and platelet accumulation in electrolytic injury mouse model with tPA‐FSNs

This experimental series in mice was conducted under protocols approved by the University of North Carolina at Chapel Hill Institutional Animal Care and Use Committee. To examine the effect of nanogels on fibrin and platelet accumulation at sites of thrombosis, a previously established electrolytic injury was created on the surface of the surgically exposed right femoral vein.^39^ Mice were anesthetized with pentobarbital (40–50 mg/kg, i.p.). Prior to injury, Alexa Fluor 647 fibrinogen and rhodamine 6G were intravenously injected (30 μg and 0.5 mg, respectively) into mice, for fibrin/fibrinogen and platelet visualization, respectively. Approximately, 3 min after injury was induced, nanogel treatment was injected intravenously. Treatment conditions included saline, 10 mg/kg FSNs, 10 mg/kg tPA‐FSNs, 10 mg/kg CS nanogels, and 10 mg/kg CS‐IgG nanogels (*n* = 4 animals per condition). In vivo imaging was conducted at the injury site continuously for 60 min (frame rate = 0.167 min) to quantify fibrin/fibrinogen and platelet accumulation over time. The experiment was terminated 60 min post‐injury. Mean fluorescent intensity plots were generated for fibrin/fibrinogen and platelet recruitment over time.

### 4.4 Examining a dose‐response of tPA‐FSN treatment in vivo in a DIC rodent model

All in vivo experiments were approved by North Carolina State University Institutional Animal Care and Use Committee and conducted in Association for Assessment and Accreditation of Laboratory Animal Care (AAALAC) international‐accredited facilities. A previously studied LPS‐induced rat model of DIC was utilized with adult male Sprague Dawley rats.^40^, ^41^ To examine a dose‐response of tPA‐FSN treatment, 0, 5, 10, or 20 mg/kg tPA‐FSNs were injected via the tail vein following the 4 h‐induction of DIC via tail‐vein infusion of LPS at 30 mg/kg. Five animals were examined per dose and five animals that did not receive LPS or tPA‐FSN infusion were used as controls. Terminal blood draws were performed to evaluate coagulation parameters, including platelet count and clot structure, 30 min after tPA‐FSN treatment. Organs were harvested to evaluate thrombi presentation in the heart, lung, kidney, and liver.

To examine thrombi presentation in the organs, MSB staining and IHC with antifibrin antibody (UC45; Genetex) and Integrin beta 3/CD61 antibody (SJ19‐09; Novus Biologicals) were conducted on sectioned tissue. Quantification of fibrin and platelets (CD61) were conducted from IHC tissue sections using ECHO labs Revolve Fluorescence Microscope. At least, three sections of tissue were imaged for each organ for each animal. Images were quantified for fibrin and platelet particle counts using ImageJ particle analysis software.

To evaluate coagulation parameters, manual platelet counts were performed using a hemocytometer and PPP was obtained from whole blood via centrifugation at 2000×*g* for 3 min, followed by a second centrifugation at 2000×*g* for 10 min. Clots formed from obtained PPP for each animal were examined using confocal microscopy as described above. Clots were made with 0.5 U/ml thrombin, 0.05 mg/ml Alexa‐Fluor 488 fibrinogen, and 5 mM CaCl_2_. At least, three z‐stack images were taken per clot. Binary z‐stack projections were quantified for fiber density (black pixels/white pixels).

### 4.5 Biodistribution of nanogels in a DIC rodent model

Biodistribution of tPA‐FSNs and unloaded FSNs in DIC animals was evaluated using fluorescently tagged particles. Here, 10 mg FSNs were incubated in 1 ml 0.2 M sodium bicarbonate pH 8.3 with 23.6 μl 750‐VivoTag‐S (5 mg/ml; Perkin Elmer) for 1 h, followed by purification via dialysis against ultrapure water for at least 48 h with three water changes. Unloaded 750‐FSNs, or 750‐FSNs loaded with tPA following the previously described method, were injected into LPS‐induced DIC rats at 10 mg/kg. Saline injected into LPS‐induced DIC rats acted as controls. Organs from previously studied biodistribution studies^29^ where saline, 750‐FSNs, or 750‐tPA‐FSNs circulated for 30 min prior to terminal blood and urine collection and organ harvest were imaged using an IVIS Xenogen In Vivo Imager using 30 s exposure time. A separate cohort of LPS‐induced DIC rats was injected with saline, 750‐FSNs, or 750‐tPA‐FSNs (10 mg/kg) and allowed to circulate for 24 h. After 24 h, organs, blood, and urine were collected from animals. Again, an IVIS Xenogen In Vivo Imager was used to scan organs at 30 s exposure time for distribution of particles. Average radiant efficiency ([p/s/cm^2^/sr]/[μW/cm^2^]) was measured for regions on interest around each organ.

### 4.6 Examining maximum tolerated dose of tPA‐FSNs in vivo

To examine the safety of high doses of tPA‐FSNs, 8‐ to 10‐week‐old male C57BL/6 mice were injected via the tail vein with 0, 50, 100, or 200 mg/kg of tPA‐FSNs in 300 μl sterile saline as a single high‐dose injection. Animals were monitored hourly for 4 h following injection for signs of severe toxicity or acute pain or distress (unable to eat, walk, or groom normally). Then, animals were monitored twice daily for 5 days postadministration for signs of toxicity, mortality, body weight, and food consumption. A bodyweight loss of 15% was used as euthanasia criteria. After 5 days, blood and organs were harvested and evaluated for organ damage, serum chemistry parameters, and coagulation parameters were performed by IDEXX BioAnalytics (North Grafton, MA).

### 4.7 Hemolysis assay

Citrated whole blood was obtained from Zen Bio (Durham, NC). Erythrocytes were isolated by centrifuging whole blood at 1500 rpm for 10 min followed by three washes in sterile saline. Isolated red blood cells were diluted 1:1 with saline to obtain 50% hematocrit stock. Concentrations of 0.1 and 1 mg/ml CS, FSN, and tPA loaded FSNs were added to tubes containing 950 μl sterile saline and 50 μl of 50% hematocrit erythrocyte solution. Particle concentrations were chosen from the in vivo human equivalent dose (0.01 mg/mg) and 100× that dosage (1.0 mg/ml).^42^ Sterile saline was used as a 0% hemolytic solution (negative control) and 0.1% Triton X was used as a 100% hemolytic solution (positive control). Solutions were incubated on a shaker at 37°C for 1 h prior to removal of intact red blood cells by centrifuging at 10,000 rpm for 5 min. The supernatant was removed and absorbance was read at 540 nm. Percent hemolysis was calculated as follows: Hemolysis rate (%) = ($A_{t}-A_{\mathrm{nc}}$)/($A_{\mathrm{pc}}-A_{\mathrm{nc}}$) × 100, where *A*_*t*_ = absorbance of test supernatant, *A*_*nc*_ = absorbance of negative control supernatant, and *A*_*pc*_ = absorbance of positive control supernatant.^43^

### 4.8 Statistical analysis

Statistical analysis was performed by using GraphPad Prism 8 (GraphPad, San Diego, CA). Fibrin binding, clot degradation assays, biodistribution studies, maximum tolerated dose, animal weight, and food consumption were analyzed via a two‐way analysis of variance with a Tukey's post hoc test using a 95% confidence interval. Dose‐response IHC quantification, platelet count, and fiber density were analyzed via a one‐way analysis of variance with a Tukey's post hoc test using a 95% confidence interval. Maximum tolerated dose hematology and chemistry parameters were analyzed via a one‐way analysis of variance with a Dunnett post hoc test using a 95% confidence interval. Outlier tests were performed on all data sets before statistical analysis. All data are presented as average ± SD.

## 5 CONCLUSIONS

The work presented here examines key translational parameters for targeted delivery of tPA for treating thrombotic complications and reducing the risks associated with systemic anticoagulants. Overall, these studies show that tPA‐FSNs are well tolerated in the body and work by targeting fibrinolytic capabilities directly to fibrin formations to lyse thrombi and correct consumptive coagulopathy.

## CONFLICT OF INTEREST

Dr. Brown is the Founder and CEO of Selsym Biotech, Inc., a startup company focused on developing fibrin‐targeted hemostatic agents.

## AUTHOR CONTRIBUTIONS

**Emily Mihalko:** Conceptualization (equal); data curation (lead); formal analysis (equal); methodology (equal); writing – original draft (equal); writing – review and editing (equal). **Kimberly Nellenbach:** Data curation (supporting); formal analysis (supporting); investigation (supporting); project administration (supporting); writing – review and editing (supporting). **Manasi Krishnakumar:** Data curation (supporting); formal analysis (supporting); investigation (supporting); writing – review and editing (supporting). **Nina Moiseiwitsch:** Data curation (supporting); methodology (supporting). **Jennifer Sollinger:** Investigation (supporting); methodology (supporting). **Brian Cooley:** Data curation (supporting); formal analysis (supporting); investigation (supporting); methodology (supporting); resources (supporting); writing – review and editing (supporting). **Ashley Brown:** Conceptualization (equal); formal analysis (equal); funding acquisition (lead); methodology (equal); project administration (lead); resources (lead); supervision (lead); writing – review and editing (lead).

## Supporting information


**Data S1.** Supporting information.

## Data Availability

The data that support the findings of this study are available from the corresponding author upon reasonable request.
