# Supplementary material for: Fibrin‐specific poly(N‐isopropylacrylamide) nanogels for targeted delivery of tissue‐type plasminogen activator to treat thrombotic complications are well tolerated in vivo
Source: Bioeng Transl Med. 2021 Dec 11;7(2):e10277. doi: 10.1002/btm2.10277 (PMC9115681; doi:10.1002/btm2.10277)
Supplement: Supplementary file 1 — Data S1. Supporting information. [file BTM2-7-e10277-s001.pdf]

Supplemental Information

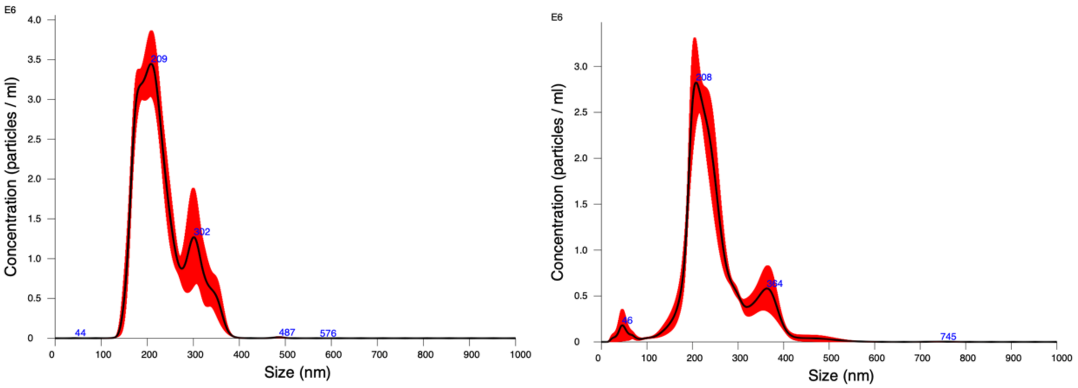

Supplemental Figure 1: Nanosight particle tracking analysis of Fibrin Specific Nanogels. Particle size distribution of hydrodynamic measurements of unloaded FSNs (left) and FSN nanogels loaded with tPA (right) were characterized via Nanosight particle tracking analysis software. At least 10^8 core and core-shell nanogels were tracked. Red error bars indicate +/- standard error of the mean.

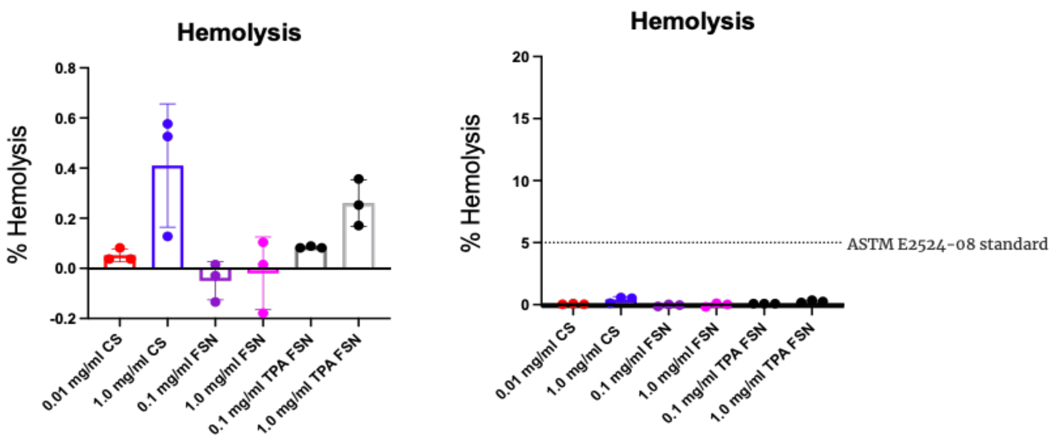

Supplemental Figure 2: Hemolysis assay with nanogels. Incubation with the human equivalent dose (HED) of 0.01 mg/mL, and 100x the HED of 1 mg/mL, does not elicit hemolysis. Unconjugated core-shell nanogels, fibrin-specific nanogels (FSNs), and tPA loaded FSNs were examined for hemolysis and all particle types at the HED and 100x HED are under the ASTM E2524-08 standard of 5% hemolysis (right).

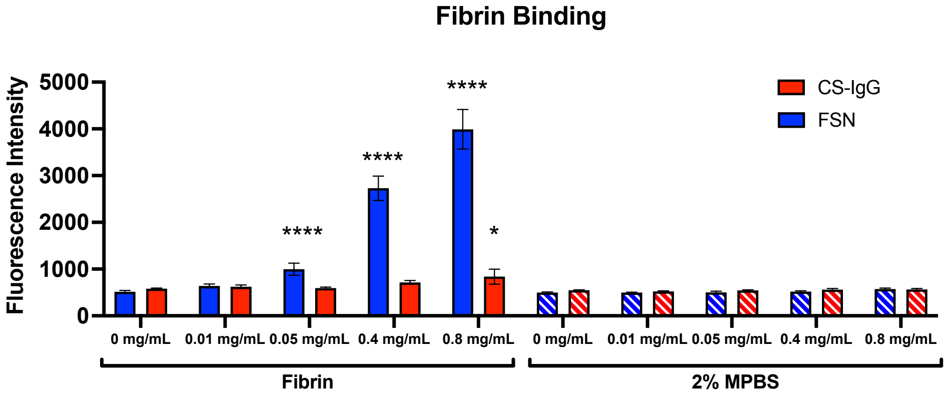

Supplemental Figure 3: Fibrin binding assay. Binding of FSNs and control CS-IgG particles at 0, 0.01, 0.05, 0.4 and 0.8 mg/mL concentrations was on fibrin coated wells or negative control 2% MPBS wells (n = 3-6/group). Mean fluorescence intensity ± standard deviation is shown. Data was analyzed via a two-way analysis of variance with a Tukey's post-hoc test using a 95% confidence interval. ****: $p<0.0001$ *: $p<0.05$.
